# ERP study on the associations of peripheral oxytocin and prolactin with inhibitory processes involving emotional distraction

**DOI:** 10.1186/s40101-019-0196-z

**Published:** 2019-05-17

**Authors:** Sayuri Hayashi, Ayami Tsuru, Fumi Kishida, Yeon-Kyu Kim, Shigekazu Higuchi, Yuki Motomura

**Affiliations:** 1 Department of Kansei Science, Graduate School of Integrated Frontier Sciences, Kyushu University, 4-9-1 Shiobaru, Minamiku, Fukuoka, 815-8540 Japan; 2 School of Design, Kyushu University, 4-9-1 Shiobaru, Minamiku, Fukuoka, 815-8540 Japan; 3 Department of Human Science, Faculty of Design, Kyushu University, 4-9-1 Shiobaru, Minamiku, Fukuoka, 815-8540 Japan

**Keywords:** Emotional distraction, Oxytocin, Prolactin, d-prime, Nogo-N2, Nogo-P3, Maternal brain, Child maltreatment

## Abstract

**Background:**

Child maltreatment is a major health and social welfare problem, with serious and longstanding consequences. Impulse control ability plays an important role in reducing the risk of child maltreatment. The aim of this study was to investigate the associations of oxytocin (OXT) and prolactin (PRL) with behavior inhibition using children’s facial expressions (angry or neutral) as emotional distractions. This may clarify a part of the neuroendocrinological mechanism that modulates impulse control ability in the context of child caregiving.

**Methods:**

Participants were 16 females who had never been pregnant. Following venous blood sampling for OXT and PRL levels, participants performed an emotional Go/Nogo task during their follicular and luteal phases to test inhibitory control ability. Behavioral performance and event-related potentials (ERPs) during the task were measured.

**Results:**

The results showed that there were significant fixed effects of OXT on behavioral performance, as measured by sensitivity (d-prime). This suggests that high peripheral OXT levels may be associated with better performance on the emotional Go/Nogo task, regardless of emotional distractors. PRL was associated with inhibitory processes as reflected by the Nogo-N2 and Nogo-P3. Particularly, high PRL levels were associated with the Nogo-N2 latency extension with the emotional distractors.

**Conclusions:**

Our findings suggest that OXT might be associated with improving behavioral performance regardless of emotional processes. It is suggested that processes related to PRL are related to premotor activities of behavioral inhibitions and emotions.

## Background

Child maltreatment is regarded as a major health and social welfare problem in high-income countries. Painful experiences in childhood such as abuse and neglect cause serious consequences for children. These experiences are associated with an increase in emotional disorders, anxiety disorders, and in the risk of suicide throughout life [1–4].

Child maltreatment may stem from functional difficulties in caregiving behaviors. For example, studies investigating parent characteristics found that difficulty with impulse control and attention-deficit hyperactivity disorder (ADHD) traits were more likely to be associated with severe child maltreatment. This suggests that maternal impulse control disability may be one of the risk factors for child maltreatment [5, 6].

Understanding the neurobiological basis for maternal caregiving will contribute to increased knowledge of how to prevent and respond to child maltreatment [7]. In most mammalian females, pregnancy and birth serve as a trigger for child caregiving behaviors such as nesting, grooming, and protection from predators. A previous review indicated that caregiving behaviors of mothers may be supported by unique brain activities that reinforce maternal behaviors, called the maternal brain [8]. The origin of the maternal behavior of humans and other mammals is likely to be the regulated by a similar mechanism. In recent years, changes in brain structure associated with pregnancy and childbirth in humans has been reported, making this theory more powerful [9, 10].

The maternal brain is influenced by hormones including oxytocin (OXT) and prolactin (PRL) [8, 11]. The secretion of OXT and PRL greatly increases with pregnancy, childbirth, and lactation. OXT is a neuropeptide whose physiological function regarding labor and lactation is well known. OXT receptors exist in brain areas related to maternal behavior, emotion, and social communication, including the medial amygdala [12, 13], ventral tegmental area, and the medial preoptic area (MPOA) [14]. Some studies suggest that OXT actually has effects on psychological and psycho-behavioral functions related to caregiving behaviors [11, 15–19]. PRL is known as a pleiotropic hormone that regulates various physiological processes such as angiogenesis, immune response, osmoregulation regulation, and reproductive behavior. It is also suggested that PRL affects the cerebral cortex and hypothalamus, and may contribute to the production of caregiving behavior in mothers [20]. OXT and PRL influence not only caregiving behavior, but are also associated with responses to emotional expressions of children and infants [17, 21–24].

Based on the above findings, maternal impulse control disability, which is one of the risk factors for child maltreatment, may disturb the maternal brain and its function. In our previous study, we reported that mothers showed enhanced behavioral inhibitory processes in the brain (observed as the larger Nogo-P3 amplitude) compared with the non-mothers. This suggests that mothers are likely to be more careful in controlling impulsive behavior than non-mothers, and it might contribute to decreasing the risk of child maltreatment [25]. However, the neuroendocrinological mechanism modulating the impulse control ability of mothers remains unclear.

Investigating the relationship between behavioral inhibitory processes and the secretion of OXT and PRL may lead to clarification of the possible mechanism modifying the impulse control ability during motherhood. In this study, an emotional Go/Nogo task was performed for testing abilities related to behavioral inhibitory processes. Children’s facial expressions were used as emotional distractors considering possible effects of children’s facial expression on caregiving behavior [16, 26]. In the emotional Go/Nogo task presenting irrelevant emotional stimuli simultaneously with the Go/Nogo cue, emotional bias was tested by investigating differences of task performance or brain activities between emotional and non-emotional condition [27]. Thus, this makes it possible to investigate the ability of inhibitory control with emotional bias from children’s facial expressions.

Event-related potentials (ERPs) calculated from electroencephalogram (EEG) are very small voltages generated in the brain in response to stimuli. ERPs can be elicited by a wide variety of sensory, cognitive, or motor events. Previous studies using ERPs methods indicated that two waveforms reflecting different functions appear at different times during the emotional Go/Nogo task. The first, known as Nogo-N2, is a negative peak potential, which appears between 200 and 300 ms from the Nogo-cue onset. The second is the Nogo-P3, which has a positive peak at approximately 400 ms from the Nogo-cue onset. Both components are predominant in the front-central region on the scalp. Many studies suggest Nogo-N2 as the index representing inhibitory processes occurred in premotor levels [28–32] and Nogo-P3 as the index representing inhibitory control of behavior [28, 32–36]. Studies using simultaneous measurement of ERPs and functional magnetic resonance imaging (fMRI) suggest that the origins of Nogo-N2 and Nogo-P3 during the emotional Go/Nogo tasks include the anterior cingulate cortex (ACC) and prefrontal areas such as orbitofrontal cortex.

This study was designed to investigate the associations of OXT and PRL with impulsivity in the context of caregiving to children excluding the effects of pregnancy and parenting experiences. Healthy females who had never been pregnant and who had no experience with child caregiving were selected as participants to rule out the effects of biological and psychological experiences from motherhood. In order to investigate the influence of fluctuation of OXT and PRL secretions, the experiments were conducted in different phases of the female menstrual cycle. It is known that OXT secretion increases during the follicular phase and ovulation phase, whereas PRL secretion increases during the ovulation phase and luteal phase. Participants participated in the experiment during the follicular and luteal phase. Peripheral OXT and PRL levels were measured. It was suggested that peripheral plasma OXT inform us of central oxytocinergic brain activity [37]. Moreover, peripheral PRL is considered the major effector within the brain [38]. Participants performed an emotional Go/Nogo task with children’s facial expressions (angry or neutral) as distractors. The association of OXT and PRL with indices of inhibitory processes, including task performance and ERPs related to inhibitory processes (Nogo-N2, Nogo-P3), were investigated. We conducted an experiment to determine whether high concentrations of OXT and PRL were associated with high impulse control ability by using children’s facial expressions as distractors.

## Methods

### Participants

Eighteen Asian women (17 Japanese and one Korean living in Japan for 27 years, mean age 27.3, SD = 4.68 years) participated in this study. They did not use oral contraceptives, had regular menstrual cycles length between 23 and 33 days, and had never been pregnant. Participants completed a questionnaire to verify that they had (1) no previous head injuries resulting in cognitive impairment, or unconsciousness lasting longer than 5 min; (2) no neurological disability, delay of cognitive development, or other disease which may possibly impair neurological and cognitive functions; (3) they did not see a doctor regularly; (4) had not visited obstetrics or gynecological clinics in the past 6 months; and (5) were not cigarette smokers or heavy caffeine drinkers, defined as drinking more than 300 mg caffeine per day [39].

Participation in the experiment was in the follicular and luteal phases, respectively. The interval of the experiment days was about 2 weeks for each participant. Menstrual cycles were estimated by the menstruation start days of the past 3 months and confirmed by basal body temperature and serum hormone concentration during the experiment days. There were significant differences between the follicular and luteal phases for OXT (*t* (15) = 3.05, *p* = 0.008) but not for PRL (*t* (15) = − 0.38, *p* = 0.704) (Table 1). Participants who showed no increase of the basal body temperature and serum progesterone concentrations during the luteal phase were excluded from the analysis. Drinking and hard-exercise were prohibited for 1 day prior to the experiment, and eating was prohibited for 2 h before the experiment. Getting a full night’s sleep the night before the experiment was also indicated.

**Table 1:** The means and SDs of basal body temperature and serum hormone concentration

Mean (SD)	Follicular phases	Luteal phases	*p* value
Basal body temperature (°C)	36.3 (0.4)	36.6 (0.4)	*0*.*008*
Serum hormone concentration			
OXT (pg/ml)	979.2 (447.2)	852.9 (349.9)	*0*.*008*
PRL (ng/ml)	18.8 (9.7)	19.9 (9.6)	0.704
Estrogen (E2) (pg/ml)	112.2 (168.9)	149.1 (122.6)	0.533
Progesterone (ng/ml)	0.3 (0.7)	6.8 (9.5)	*0*.*017*
Cortisol (μg/dl)	7.5 (3.4)	6.6 (2.6)	0.349

Data are mean (SD). Significant *p* values are in italic

Informed written consent was obtained from all participants as approved by the Ethics Committee of Kyushu University (Approval Number 276).

### Experiment task

**Fig. 1:**
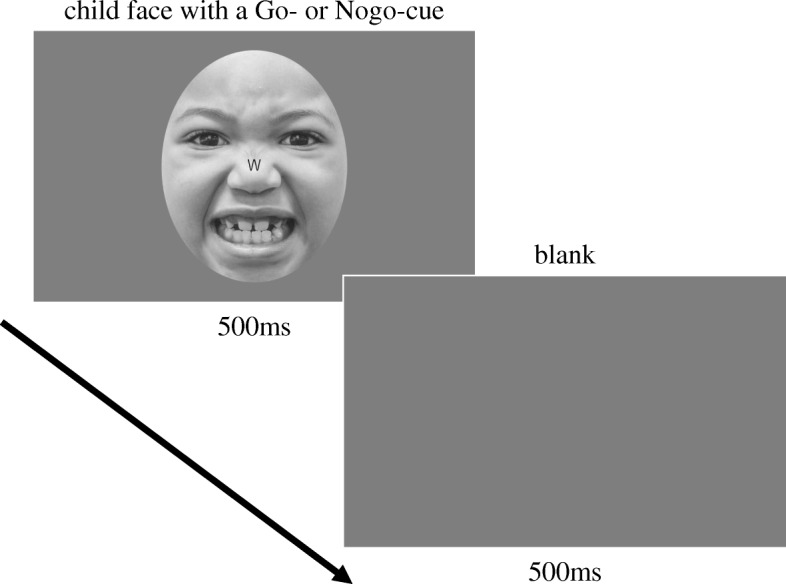
Example of a trial of the emotional Go/Nogo task. The figure shows an example of a trial of the emotional Go/Nogo task in this study. The task included four trial types (angry Go, neutral Go, angry Nogo, and neutral Nogo)

The task contained four trials: angry-Go trials (35%), neutral-Go trials (35%), angry-Nogo trials (15%), and neutral-Nogo trials (15%). Each trial started with the presentation of a child’s face (angry or neutral) and an alphabet cue (Go-cue or Nogo-cue) for 500 ms (Fig. 1). The Go- or Nogo-cue was located in a central line between the child’s eyes. Afterward, a gray blank screen was presented for 500 ms. The participants had to respond as fast as possible with their index finger to the picture with Go-cue but had to withhold a response to the picture with Nogo-cue. A block including 240 trials was carried out four times. The order of trials was randomized within each block.

The Child Affective Facial Expression (CAFE) set [40] was used for the children’s facial pictures. Twenty-four pictures of 12 children’s angry and neutral faces (six boys and six girls aged 3 to 6 years old) were selected. The race of the children was taken into consideration when selecting them to reduce any possible effects of ethnic type. The final selection included two Asians, three Caucasians, four Africans or African Americans, and three people of other races. The pictures were cropped to an elliptical shape and gray-scaled, and the brightness was unified to reduce influences from various factors as children’s hairstyle and physical visual information. The Go- or Nogo-cues were represented by the letters M or W, which were placed between the eyes in each picture. Half of the participants were instructed M and W were the Go- and Nogo-cues, respectively. The other half of participants used W and M as the Go- and Nogo-cues, respectively. A visual angle of each picture was approximately 4° × 3°.

The task required about 20 min including practice. Before starting the task, participants were told they would be given a gift card worth 500 yen if their performance was higher than the mean score of all participants. Following the emotional Go/Nogo task, an automatic facial mimicry task and 5-min rest time were implemented. The experimental task was implemented using Presentation software (Neurobehavioral Systems, Inc., USA).

### Behavioral data analysis

Go trials with a button press in the interval of 100 to 1000 ms from the Go-cue presentation were identified as correct-Go trials (HIT), and other Go trials were coded as incorrect-Go trials. Results confirmed that there were no Go trials with responses during 0 to 100 ms after Go-cue onset. A Nogo trial with no response from 0 to 1000 ms from the Nogo-cue presentation was identified as correct-Nogo trials, and Nogo trials with responses during this time were coded as error-Nogo trials (FALSE ALARM).

The detection sensitivity (d-prime) and response bias (β) were estimated to describe the task performance following the signal detection theory measures [39]. Formulas for d-prime and β are the following:

$$ \mathrm{d}-\mathrm{prime}=\mathrm{Zhit}-\mathrm{Zfalse}\ \mathrm{alarm} $$

$$ \upbeta =N\ \left(Z\mathrm{hit}\right)/N\ \left(Z\mathrm{false}\ \mathrm{alarm}\right), $$

where Zhit is the z-transformed rate of HIT in all Go trials, Zfalse alarm is the z-transformed rate of false alarms, and *N*(*Z*) represents the normal density functions (the normalized normal distributions) of *Z*.

### Psychophysiological recording and data analysis

The experiment was conducted in a shielded room for EEG recording at Kyushu University.

Following adequate verbal and written explanation of the experiment, 10 ml of venous blood was drawn before each EEG investigation to assess OXT and PRL levels. As additional indicators of the menstrual cycle, determinations of estradiol, progesterone, and cortisol were also assessed. The timing of the blood sampling was unified within each participant. Determination of OXT was performed using a commercial OXT ELISA kit (Enzo Life Sciences, Ann Arbor, MI) and determination of other hormones was analyzed by electro-chemiluminescence immunoassay (ECLIA) technology.

The EEG was conducted with a 64-channel EEG measuring system (64-channel HydroCel GSN, Net Amps 200 64-channel EEG Amplifier, and Net Station, ver. 4.1.2; Electrical Geodesics Inc., USA). A reference electrode was located at Cz in the International 10–20 system. The data were sampled by a 500 Hz with hardware filter (0.01 to 100 Hz). The impedance of electrodes was kept at 60 kΩ or less.

All EEG pre-processing was conducted using MATLAB 2017a (the MathWorks, Inc., Natick, MA, USA) and EEGLAB ver 14.1.1 [41]. Raw EEG data were filtered by a FIR band-pass filter (0.5–40 Hz; transition band width 1 Hz). The data were divided as epochs from − 500 to 1000 ms. The presentation of facial pictures with the Go- or Nogo-cue was used as the onset. Bad epochs and channels were automatically rejected using functions of EEGLAB. The data were re-referenced to the common average reference [42] and subjected to independent component analysis. Independent components representing eye-blinks or eye-movements were manually rejected based on the topographical map and the frequency spectrum [43].

**Fig. 2:**
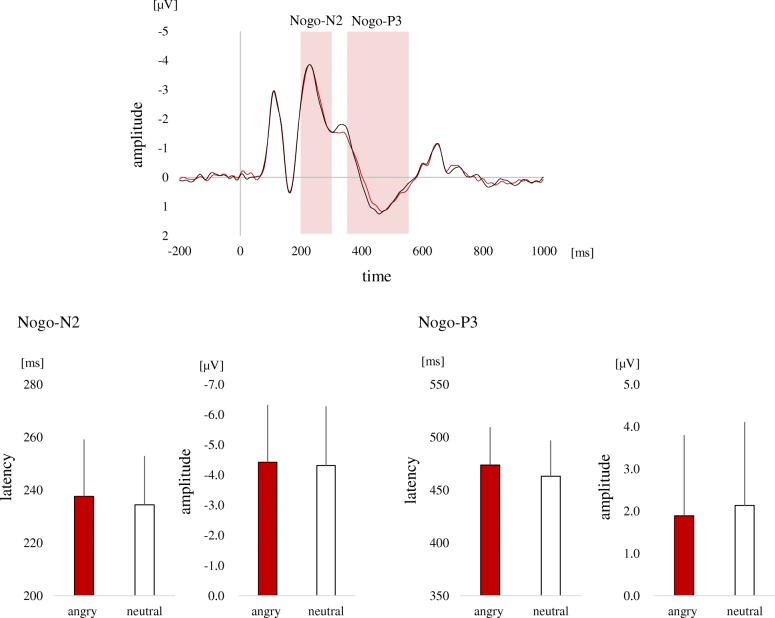
ERP waveforms and Nogo-N2 and Nogo-P3 in the fronto-central region. The figure above shows ERPs (grand average waveforms) obtained from correct-Nogo trials. The red and the black lines indicate ERPs for the angry and neutral condition, respectively. The below shows amplitudes and latencies of the Nogo-N2 and Nogo-P3. The red and white bars indicate ERPs for the angry and neutral condition, respectively

The event-related potentials (ERPs) were calculated within each electrode, participant, condition, and experiment day. The baseline was set from − 200 to 0 ms. ERPs were averaged across the fronto-central region, thought to reflect brain activities related to the interaction between behavior inhibition and emotion (Fig. 2). A data set from a participant was excluded from the analysis because the participant had fewer than the number of trials needed for averaging (less than 20 trials). The data from the incorrect-Go and -Nogo trials were also rejected for the same reason. In addition, this study focused on inhibitory processes, thus correct-Nogo responses were used for the following analysis. Nogo-N2 and Nogo-P3 were defined as the negative peak potential between 200 and 300 ms and the positive peak potential between 350 and 550 ms after the Nogo-cue presentation in the fronto-central region, respectively. The peak amplitudes and latency of the Nogo-N2 and Nogo-P3 were calculated (Fig. 2). The mean number of trials under angry- and neutral-Nogo was 80.1 and 83.6, respectively.

In addition to the EEG, facial electromyography (facial EMG) from left corrugator supercilii, orbicularis oculi, zygomaticus major, and levator labii surperioris was also recorded by bipolar methods with a biosignal amplifer system (PolymateII AP1532, Miyuki Giken Co., Ltd., Japan). This study focused on the inhibitory processes in the brain, so the data of facial EMG is excluded from this report.

### Statistical analysis

A liner mixed model (LMM) was used to estimate the associations of OXT, PRL, and facial expression (angry or neutral) with behavioral and EEG data including d-prime, beta, and latency, as well as amplitudes of Nogo-N2 and Nogo-P3. The model included the fixed effects of OXT, PRL, facial expressions, and the interaction of these factors with the participants as a random-intercept.

This study focuses on the associations of peripheral oxytocin and prolactin with inhibitory processes involving emotional distraction. Thus, menstrual cycle (follicular or luteal phase) and its interaction with facial expressions was included as an additional and no-interested fixed effect in the LMM. In addition, considering the effects of circadian rhythm, the number of the trials, and the ovarian hormones (estrogen and progesterone) on the menstrual cycle, the LMM also included experiment time (morning, afternoon, evening, or night), day (day 1 or day 2), estrogen, and progesterone as additional fixed effects. For the analysis of ERPs, the number of trials also added the LMM as the fixed effects. It is thought that estrogen and progesterone may associate with OXT, PRL, and emotion. Therefore, the interaction term with OXT, PRL, and facial expression was also included in the model. These additional fixed effects were rejected using the backward stepwise methods based on the Akaike’s information criterion (AIC). When there was a significant difference between the model after the stepwise and the initial model selection, the model after conducting the stepwise method was adopted.

The fixed effects of OXT, PRL, and facial expressions were tested with Kenward-Roger’s method [44] after the model selection using the backward stepwise methods. The fixed effect of the menstrual cycle was also added to the statistical significance tests in order to confirm the influence of the fluctuation associated with the menstrual cycle. Since a relationship between the menstrual cycle and emotional process was suggested, the interaction term of the menstrual cycle and facial expressions was also added to the test. Finally, the significance test included nine fixed effects; OXT, PRL, facial expressions and their interactions (OXT × facial expressions, PRL × facial expressions, OXT × PRL, OXT × PRL × facial expressions), menstrual cycles, and interaction terms of menstrual cycles and facial expressions. If the LMM model adopted by the stepwise method did not contain some of the target terms, the terms were excluded from the test. The test was performed following the false discovery rate (FDR) control method [45]. Significant alpha levels were less than 0.05. All categorical data were deviation coded (and converted to dummy variables when necessary); the continuous data were standardized prior to entering the analyses. All statistical analysis was conducted using the lme4 version1.1-18-1 [46], and lmerTest version3.0-1 [47] for R. In order to illustrate effects of the fixed effects, the effects ver 4.0-3 [48] was also used.

## Results

### Behavioral results

The mean (SD) of the sensitivity (d-prime), response bias (β), and response times for correct-Go were 1.20(0.48), 1.23(0.59), and 393.1(35.7) ms, respectively. Table 2 shows the details of linear mixed model (LMM) estimates of the OXT, PRL, facial expressions, and menstrual cycle for the d-prime and β. With respect to the d-prime, there was a main effect of OXT (*t* (28.9) = 4.77, *p* < 0.001), and as the OXT level was higher, the d-prime increased. For the β, there was no significant fixed effects of OXT, PRL, facial expressions, nor any interaction between these variables.

**Table 2:** Linear mixed model (LMM) estimates of the OXT, PRL, facial expressions, and menstrual cycle for the d-prime and β

	d-prime			β		
	Estimate	std. error	*t* value	Estimate	std. error	*t* value
OXT	1.50	0.31	4.77***	0.76	0.36	2.06
PRL	0.68	0.46	1.46	1.13	0.59	1.90
facial expressions	− 0.40	0.23	−1.71	0.006	0.38	0.01
OXT × facial expressions	− 0.22	0.26	− 0.87	− 0.14	0.42	− 0.34
PRL × facial expressions	− 0.14	0.41	− 0.34	− 0.33	0.67	− 0.49
OXT × PRL	0.62	0.76	0.86	1.69	0.98	1.72
OXT × PRL × facial expressions	− 0.26	0.69	− 0.37	− 0.24	1.14	− 0.21
Menstrual cycle	− 0.01	0.26	− 0.05	0.07	0.36	0.21
Menstrual cycle × facial expressions	0.21	0.27	0.78	0.04	0.44	0.09

∗∗∗*p* < 0.001 (FDR corrected)

### ERPs during the emotional go/Nogo task

**Fig. 3:**
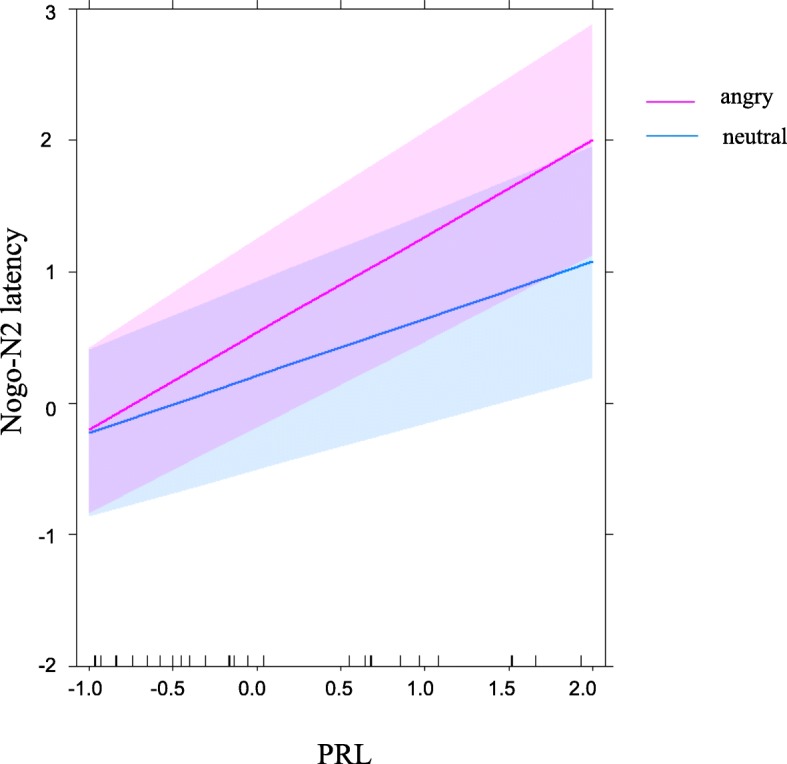
PRL effects on the Nogo-N2 latency. Predictions of the LMM estimates of the standardized Nogo-N2 latency. Colored bands represent 95% confidence intervals based on the model. The red and blue lines indicate standardized Nogo-N2 latency for the angry and neutral condition, respectively

Table 3 shows the details of LMM estimates of the OXT, PRL, facial expressions, and menstrual cycle for the Nogo-N2 and Nogo-P3. For the Nogo-N2 latency, there was a main effect facial expression (*t* (27.6) = 3.03, *p* = 0.030). The Nogo-N2 latency increased for angry facial expressions compared with neutral expressions. Furthermore, there was an interaction between PRL and emotion (*t* (27.6) = 2.64, *p* = 0.030). As the PRL levels increased, the Nogo-N2 latency extension of the angry condition was emphasized (Fig. 3). There was also an interaction between OXT and PRL (*t* (36.6) = 2.64, *p* = 0.030). The high concentration of both OXT and PRL was related to the extension of the Nogo-N2 latency. For the Nogo-N2 amplitude, there was no significant fixed effects of OXT, PRL, facial expressions, or interaction between these variables.

**Table 3:** Linear mixed model (LMM) estimates of fixed effects of the OXT, PRL, facial expressions, and menstrual cycle for the Nogo-N2 and Nogo-P3

	Latency			Amplitude		
	Estimate	std. error	*t* value	Estimate	std. error	*t* value
Nogo-N2						
OXT	0.43	0.23	1.83	− 0.05	0.34	− 0.16
PRL	0.43	0.20	2.15	− 0.98	0.60	−1.63
Facial expressions	0.32	0.10	3.03*	− 0.14	0.23	− 0.61
OXT × facial expressions	− 0.04	0.11	− 0.37	− 0.04	0.22	− 0.18
PRL × facial expressions	0.30	0.11	2.64*	0.21	0.38	0.55
OXT × PRL	0.67	0.25	2.63*	− 2.33	0.99	− 2.35
OXT × PRL× facial expressions	0.03	0.11	0.33	− 0.005	0.69	− 0.008
Menstrual cycle				0.66	0.25	2.63
Menstrual cycle × facial expressions				0.12	0.24	0.49
Nogo-P3						
OXT	0.11	0.32	0.34	− 0.35	0.18	− 1.86
PRL	0.46	0.16	2.82*	0.32	0.10	3.15**
Facial expressions	0.33	0.11	2.87*	− 0.13	0.03	− 3.30**
OXT × facial expressions	0.08	0.14	0.62	0.08	0.04	1.71
PRL × facial expressions	0.02	0.11	0.17	0.001	0.04	0.04
OXT × PRL	0.10	0.14	0.68	0.02	0.07	0.35
OXT × PRL × facial expressions	− 0.18	0.12	− 1.58	0.008	0.04	0.21
Menstrual cycles				0.08	0.04	1.71
Menstrual cycle × facial expressions				0.01	0.04	0.04

∗*p* < 0.05; ∗∗*p* < 0.01 (FDR corrected)

For the Nogo-P3 latency, there was a main effect of PRL and facial expression (PRL: *t* (41.6) = 2.82, *p* = 0.025, facial expression: *t* (33.5) = 2.87, *p* = 0.025). As the PRL levels increased, the Nogo-P3 latency increased. The Nogo-P3 latency also increased for angry facial expressions compared with neutral expressions. For the Nogo-P3 amplitude, there was a main effect of PRL and facial expression (PRL: *t* (42.0) = 3.15, *p* = 0.010, facial expression: *t* (30.2) = − 3.30, *p* = 0.010). As the PRL levels increased, the Nogo-P3 amplitude was larger. The Nogo-P3 amplitude was smaller for angry expressions compared with neutral expressions.

## Discussion

The aim of this study was to investigate OXT and PRL associations with behavior inhibition using children’s facial expressions (angry or neutral) as emotional distractors, to clarify specific neuroendocrinological mechanisms modulating impulse control ability in child caregiving. The experiments were conducted in the follicular and luteal phases of the menstrual cycles. The effects of the menstrual cycle were statistically excluded using the LMM methods. The results indicated that OXT was associated with behavioral performance measured by the sensitivity (d-prime) and response bias (β), whereas PRL was associated with inhibitory processes reflected by the Nogo-N2 and Nogo-P3. These findings indicate that OXT and PRL might have different relationships with the inhibitory processes involving emotional distractions.

There was a main effect of OXT in the association of OXT with sensitivity (d-prime). As the OXT level increased, the d-prime increased. This suggests a possibility that the high peripheral OXT level may be associated with good performance on the emotional Go/Nogo task. However, there was no significant interaction between the facial expressions and OXT. Previous studies have described the effects of administrating OXT on emotions via the modulation of neural activity in emotional regions of the brain such as the amygdala [23, 49, 50]. Based on these studies, we expected a significant interaction between OXT and facial expressions on the inhibitory processes via the modulation of processing children’s angry faces as the distractor. Contrary to our expectations, the results suggested that changes in OXT levels related to menstrual cycles might be related to controlling impulsive behaviors regardless of emotional processes. It is suggested that large differences in OXT concentrations between previous studies and this study might have caused the contradictory result. A review of intranasal OXT indicated that the peak plasma concentration of OXT would exceed 1400 pg/mL by 24 IU OXT [51], which is about 1.5 orders of magnitude higher than the plasma OXT concentrations in this study (Table 1). Intranasal administration of OXT raises peripheral concentrations to supraphysiological levels, which might have differentially affected the studies [51]. As far as we know, this is the first study to demonstrate an association between slight, intrinsic fluctuations of OXT, and the control of impulsive behavior, rather than the distractive effects of children’s angry faces. In a previous study on the relationships between OXT and impulsive control ability, serum OXT levels showed a negative correlation with impulsivity [52]. This is consistent with the results of this study showing the effects of OXT on behavioral performance. Furthermore, those results suggest that as the OXT levels increase, the impulsivity is higher regardless of the emotional processes. Therefore, our result on the association between high OXT and good behavioral performance might reflect the effects of OXT on attentional processing or its associations with impulse control ability.

Despite those results, we could not confirm the significant fixed effects of OXT on the Nogo-N2 and Nogo-P3. Previous studies showed that the Nogo-N2 and Nogo-P3 reflect the inhibitory processes during behavioral inhibitory tasks including the emotional Go/Nogo task [33, 53–57]. However, it is also known that performing the emotional Go/Nogo task needs not only brain activities reflected in the Nogo-N2 and Nogo-P3 such as inhibitory processes at the ACC and orbital frontal cortex [34, 35, 56, 58–60] but also other regions including subcortical regions, like the amygdala, ventral striatum, and anterior insula [61–63], and some prefrontal cortices such as ventral-lateral prefrontal cortices and medial prefrontal cortex [61, 64–68]. The results of this study may possibly reflect the associations of OXT with task performance via processing not reflected in the Nogo-N2 and Nogo-P3.

On the other hand, the significant interaction between the PRL and facial expression was observed in the Nogo-N2. Regarding the Nogo-N2, as the PRL levels increased, the Nogo-N2 latency extension was emphasized, especially in the angry condition (Fig. 3). This suggests the possibility that high peripheral PRL levels might be associated with facilitating emotional effects of angry faces in the Nogo-N2.

The Nogo-N2 is known as an ERP component reflecting inhibitory processes. It is thought that these processes include emotional process and mainly originate from the ACC. The activities of the ACC are thought to be influenced by facial expression stimulus via modulation from other regions, which play an important role in emotional processes such as the amygdala [35]. It has been demonstrated that the ACC has a strong connection with the amygdala, ventral striatum, and orbital frontal cortex [69, 70]. It has also been reported that these three regions were involved in processes related to interactions between behavioral inhibition and emotion [71–73]. The influence of facial expressions may be transmitted through the amygdala to remote areas including the ACC [74, 75], and some of the inhibitory processes of ACC affected by the facial expression stimulus may be observed as the Nogo-N2 [34, 35]. For the anger condition, the influence of facial expressions was evident and it would cause delayed latency of the Nogo-N2.

It has been experimentally shown that the Nogo-N2 is functionally different from the Nogo-P3, and the Nogo-N2 may reflect inhibitory processing followed by the behavior, response, or motor inhibition processes. It is sometimes referred to as the inhibitory processes at the premotor level [53, 54, 57]. A recent study reported that the origin of the Nogo-N2 also included the supplementary motor cortex and cingulate cortical motor cortex, which indicate a theory that the Nogo-N2 could reflect premotor activity giving rise to the premotor (Bereitschaftspotential) component [76]. Considering this, the result of the association of PRL with the Nogo-N2 may suggest that the high peripheral PRL levels may be associated with facilitating the emotional effects of the angry faces on the processes in the premotor level reflected by the Nogo-N2. This may possibly include inhibitory processes mainly originated in the ACC and affected by emotional information via modulation from some brain regions such as the amygdala.

PRL is a hormone synthesized and secreted primarily by the anterior pituitary, and it is also secreted by many peripheral tissues such as the mammary gland. Its receptors are expressed in the pituitary gland and peripheral tissues and are also consistently detected in the brain regions such as the cerebral cortex, the olfactory bulb, the hypothalamus, the hippocampus, and the amygdala [20]. A recent rodent study focused on the maternal brain experimentally suggested that PRL may be important for neural activities that facilitate maternal caregiving. In the study, neurons directly affected by PRL were visualized in suckled mother mice. The results showed that the PRL may play important role in several brain regions, including the lateral septum, medial amygdaloid nucleus, subparafascicular area, caudal periaqueductal gray, dorsal raphe, lateral parabrachial nucleus, nucleus of the solitary tract, and the periventricular, medial preoptic, paraventricular, arcuate, and ventromedial nuclei of the hypothalamus [77]. Other experiments chronically administering PRL to pregnant rats with ovariectomy suggest that PRL may inhibit thalamic relay nuclei, which integrate signals of stress perception before they are transmitted to layers of upper sensory cortex [38, 78]. Another study, based on similar mechanisms in other mammals, has suggested that PRL is likely to play a significant role in altered emotionality in humans [22]. These studies support the idea that PRL may contribute to the production of caregiving behavior of mothers by affecting brain regions including emotional areas like the amygdala. Such effects of PRL might possibly affect the results of this study.

Apart from the context of childcare, a study of dietary dopamine depletion showed that the peripheral prolactin levels modulated by dopamine depletion manipulation may be associated with the neural activation to face trustworthiness in the amygdala, medial orbital frontal cortex, and ventral medial prefrontal cortex. This suggests the possibility that PRL levels may be associated with the sensitivity to the social stimuli in these brain areas [79]. Interestingly, these regions affect the ERPs during the emotional Go/Nogo task. As mentioned above, emotional moderation by the amygdala may be reflected by the Nogo-N2 and some studies estimate that the origin of the Nogo-N2 includes the orbital frontal cortex [35, 56, 58–62, 74, 75]. Regarding the ventral medial prefrontal cortex, it is reported that high-risk behavior with an emotional Go/NoGo task was related to the orbital frontal cortex and ventromedial prefrontal cortex [80]. Other studies reported that there were interactions between the ventromedial prefrontal cortex and amygdala during the inhibitory task [81]. Considering these results, the effects of PRL may possibly affect the Nogo-N2 via altered brain activities in these regions, which might be related to processes for facial expression as social stimuli.

The interaction term of OXT and PRL was significant for the Nogo-N2 latency, and high concentrations of OXT and PRL were related to the extension of the Nogo-N2 latency. This suggests that inhibitory processing reflected in the Nogo-N2 latency might be extended when concentrations of OXT and PRL are high. Research on caregiving for children has suggested that OXT and PRL are involved in processes related to children’s face and caregiving behaviors [21, 82–84]. One study has investigated the effects of PRL on the behavioral and neuroendocrine responses related to maternal behavior. In the study, lactating rats performed maternal behavior and stress tests. The rats with inhibited expression of PRL receptors in the brain showed not only impaired maternal behaviors such as increased latency of starting to retrieve their young but also large stress responses that are modulated by the OXT system. This suggests that the PRL system might influence not only promoting maternal behavior but also facilitating the OXT system that decreases responses to physical and emotional stressors [85]. Similar interactions between OXT and PRL are also suggested in a review of studies on rodent maternal brains. This interaction is thought to be related to reducing stress responses of mothers including anxiety associated with child caregiving [22]. These studies suggest the possibility that maternal caregiving anxiety is reduced by the OXT system under high OXT and PRL concentrations. Another study using the ERP method demonstrated that threat-related thoughts might also influence the Nogo-N2, by suggesting that aversive feedback might induce inhibitory processes reflected by the Nogo-N2 via increased threat-related conflict monitoring. Threat-related thoughts during behavioral inhibitory tasks might facilitate inhibitory processes reflected by the Nogo-N2 [86]. Reduced anxiety via the OXT system under high-PRL might reduce threat-related thoughts, which could extend the Nogo-N2 latency.

The PRL level was also related to the prolongation of Nogo-P3 latency and the large Nogo-P3 amplitude, irrespective of facial expressions. There are few studies on the relationship between the behavioral inhibition and PRL. Future studies, such as investigation of the role of PRL in the regions related to behavioral inhibition and the relation with peripheral PRL, may be necessary. In addition, significant fixed effects of PRL were observed only in the ERPs, which invites caution about the interpretation.

Our results indicated that OXT could be associated with controlling impulsive behaviors rather than directly affecting emotional processes. In the studies on ADHD, high ADHD traits were associated with low peripheral OXT levels [52]. Other studies reported that maternal impulse control disability may be one of the risk factors for child maltreatment with participants diagnosed with ADHD [5, 6]. Perhaps low OXT levels may cause increased risk of child maltreatment because of the diminished ability to control impulsive behaviors. In other words, OXT may assist with controlling impulsive behaviors, regardless of the social context, which is important for child caregiving.

The fixed effects of PRL were observed in the Nogo-N2 and Nogo-P3 premotor and motor inhibitory processes, respectively. Emotional effects of the angry facial expression were associated with the peripheral PRL levels in the premotor processes reflected by the Nogo-N2 (Fig. 3). Therefore, it is possible that PRL is associated with making allocations for facial recognition and behavioral inhibition when prioritizing sensitivity to children’s expressions without reducing behavioral performance.

Our findings might be useful in suggesting a new role of OXT and PRL. Nevertheless, there are certain limitations to this study which mandates further study. First, based on other studies, the peripheral OXT and PRL levels were used as indices of OXT and PRL [37, 38]. Also, only healthy women that had never been pregnant nor had experienced caregiving to children were selected as participants. It is suggested that the effects of OXT and PRL should be tested using other types of participants including biological mothers, as well as women that are perpetrators of child abuse.

## Conclusions

In conclusion, the current findings suggest that OXT and PRL might be associated with inhibitory processes related to children’s facial expressions as emotional distractions. OXT might be associated with improving behavioral performance regardless of accompanying emotional processes. PRL might be associated with premotor activities interacting between processes related to behavioral inhibition and emotions. The association of OXT and PRL, the secretions of which greatly increase with pregnancy, childbirth, and lactation, with inhibitory processes might reflect mechanisms for reducing the risk of child maltreatment by biological mothers.

## Abbreviations

ACC: Anterior cingulate cortex
AIC: Akaike’s information criterion
ANOVA: Analysis of variance
EEG: Electroencephalogram
ERPs: Event-related potentials
FDR: False discovery rate
LMM: Liner mixed model
OXT: Oxytocin
PRL: Prolactin
